# Experimental and Modeling Analysis of Polypropylene Fiber Reinforced Concrete Subjected to Alkali Attack and Freeze–Thaw Cycling Effect

**DOI:** 10.3390/ma17184529

**Published:** 2024-09-14

**Authors:** Yuxiang Huang, Yongcheng Ji, Jingchen Wang, Zihao Wang, Bosong Yu, Siyu Zhang

**Affiliations:** 1 College of Aulin, Northeast Forestry University, Harbin 150040, China; rickesthyx@nefu.edu.cn (Y.H.); jingchenwang@nefu.edu.cn (J.W.); wzh@nefu.edu.cn (Z.W.); 2022214799@nefu.edu.cn (B.Y.); 2 College of Civil Engineering and Transportation, Northeast Forestry University, Harbin 150040, China; zsy7523@163.com

**Keywords:** ASR, polypropylene fiber concrete, freeze–thaw cycle, Weibull distribution, parallel bar system

## Abstract

The durability of concrete materials in harsh environmental conditions, particularly in cold regions, has garnered significant attention in civil engineering research in recent years. Concrete structures in these areas are often damaged by the combined effects of alkali–silica reaction (ASR) and freeze–thaw cycles, leading to structural cracks and significant safety hazards. Numerous studies have demonstrated that polypropylene fiber concrete exhibits excellent crack resistance and durability, making it promising for applications in cold regions. This study elucidates the impact of alkali content on concrete durability by comparing the mechanical properties and durability of different alkali–aggregate concretes. The principal experimental methodologies employed include freeze–thaw cycle experiments, which examine patterns of mass loss; fluctuations in the dynamic modulus of elasticity; and changes in mechanical properties before and after freeze cycles. The findings indicate that increased alkali content in concrete reduces its strength and durability. At 100% alkali–aggregate content, compressive strength decreases by 35.5%, flexural strength by 32.9%, mass loss increases by 35.85%, relative dynamic elastic modulus by 39.4%, and residual strength by 97.28%, indicating higher alkali content leads to diminished durability. Additionally, this paper introduces a constitutive damage model, validated by a strong correlation with experimental stress–strain curves, to effectively depict the stress–strain relationship of concrete under varying alkali contents. This research contributes to a broader understanding of concrete durability in cold climates and guides the selection of materials for sustainable construction in such environments.

## 1. Introduction

Concrete, a material extensively utilized globally, is employed in substantial quantities across diverse types. Owing to its pervasive application, numerous researchers have dedicated efforts to examining its engineering properties [1,2,3,4]. The evolution of modern civil engineering has catalyzed a growing demand for innovative concrete variants, necessitating improved properties such as strength, toughness, crack resistance, and durability [5,6]. Concrete structures located in cold regions are primarily affected by the dual influence of the region’s inherent cold characteristics and the progressively increasing service life. Over extended periods, these structures experience the synergistic effects of freeze–thaw cycles and ASR [7,8,9,10]. The load-bearing capacity of structures can be significantly compromised, adversely affecting the longevity and safety of concrete structures. This reduction may hinder achieving their anticipated service life, resulting in significant economic losses and heightened safety risks. To tackle these challenges, researchers have investigated multiple strategies to enhance concrete structures’ structural strength and overall performance. One practical approach involves incorporating fiber materials into the concrete matrix, which helps to overcome its inherent limitations. The incorporation of these fibers significantly enhances the performance of concrete by effectively constraining the matrix and preventing crack propagation [11,12,13,14,15]. Among the numerous fiber additives available, polypropylene fiber (PPF) is especially notable as a widely used synthetic fiber in construction materials. Derived from the polymerization of propylene, this linear polymer exhibits several beneficial properties, including low weight, high strength, exceptional toughness, and corrosion resistance [16,17,18]. These attributes contribute to the widespread application of PPF across various sectors, such as the chemical industry, energy, textile, environmental protection, and construction [19,20,21,22]. Recent studies have demonstrated that polypropylene fibers substantially enhance concrete’s compressive and tensile strengths and improve its toughness and crack resistance. These fibers effectively reduce the likelihood of micro-crack formation and the subsequent propagation of cracks within the concrete. Such findings provide crucial insights for improving the durability and safety of concrete in real-world engineering applications [23,24,25,26,27,28,29].

As a novel variant of concrete, it is crucial to examine the durability of polypropylene fiber-reinforced concrete under extreme environmental conditions. Research conducted by various scholars has indicated that including polypropylene fibers can improve the freeze–thaw cycle resistance of concrete. Dong and Gao [30] studied the impact of fiber type and content on the frost resistance of airport pavement concrete. It was found that concrete reinforced with PPF exhibits superior frost resistance compared to steel fiber-reinforced concrete. Optimal enhancement in frost resistance was observed by adding 1% PPF (by volume content) to the concrete mix. Nam et al. [31] conducted a comparative study on the effects of PPF and polyvinyl alcohol fiber on concrete’s freeze–thaw cycle resistance. The findings indicated that polypropylene PPF-reinforced concrete exhibits a lower rate of mass loss and reduced decreases in dynamic elastic modulus and strength compared to plain concrete. Additionally, the resistance of PPF-reinforced concrete to freeze–thaw cycles was significantly influenced by the dispersion of the fibers within the mix and the bonding performance between the fibers and the cementitious materials. It suggests that optimizing fiber distribution and improving interfacial bonding can further enhance the durability of concrete in environments subjected to freeze–thaw conditions. Atis and Karahan [32] investigated the freeze–thaw resistance of polypropylene fiber-reinforced concrete. They discovered that the freeze–thaw resistance of PPF-reinforced concrete was marginally improved compared to plain concrete. Notably, the freeze–thaw resistance of PPF-reinforced concrete can be significantly augmented by incorporating fly ash as an active mineral admixture. Jun et al. [33] investigated the durability indices of rubber concrete formulated with macrosynthetic polypropylene fibers, focusing on aspects such as drying shrinkage, ASR expansion, and frost resistance. They concluded that PPF-reinforced rubber concrete exhibits enhanced durability compared to plain concrete.

Researchers found that PPF can also be utilized to improve the ASR resistance of concrete [29]. Daria et al. [34] reveal that incorporating polypropylene fibers into cement-based systems can diminish the expansion caused by ASR. This expansion reduction is on par with the effects achieved using an equivalent dosage of steel fibers. Gao et al. [35] explored the effects of adding polypropylene fiber and recycled tire rubber powder to concrete. Their findings suggest that polypropylene fibers can effectively inhibit crack propagation caused by ASR through their inherent bridging effect. However, many existing studies have primarily focused on performance changes under single-variable conditions. Concrete often deteriorates due to the combined effects of multiple environmental factors. These interactive conditions can significantly impact the performance of PPF-reinforced concrete, highlighting the need for more comprehensive research that considers the simultaneous influence of various environmental stresses on concrete durability and performance.

The ASR and freeze–thaw cycles are crucial factors in determining the durability of concrete. PPF-reinforced concrete, a material with significant potential in the construction industry, has yet to be the focus of studies investigating the synergistic impact of ASR and freeze–thaw cycles on its durability. Therefore, this paper contributes to the theoretical understanding of these effects and has practical implications for using polypropylene fiber concrete in cold areas. The experimental approach involves using four different alkali aggregates, altering the ASR degree by varying the mix proportions. A constitutive model based on the Weibull theory is proposed to describe the effect of ASR with different alkali–aggregate content on the mechanical properties of polypropylene fiber concrete. The findings also provide insights into the patterns of mass loss and dynamic modulus of elasticity in concrete with varying alkali contents over freeze–thaw cycles, as well as the changes in mechanical properties of concrete before and after the cycles, which can guide the design and use of polypropylene fiber concrete in extreme environmental conditions.

## 2. Materials and Methods

### 2.1. Materials

**Figure 1:**
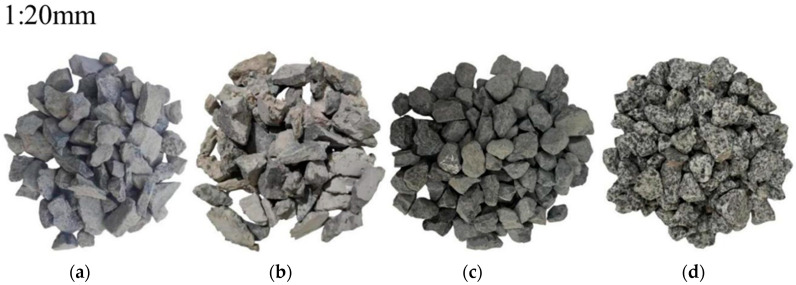
Aggregate used in the experiment: (**a**) crushed stone; (**b**) recycled concrete; (**c**) basalt; (**d**) granite.

**Figure 2:**
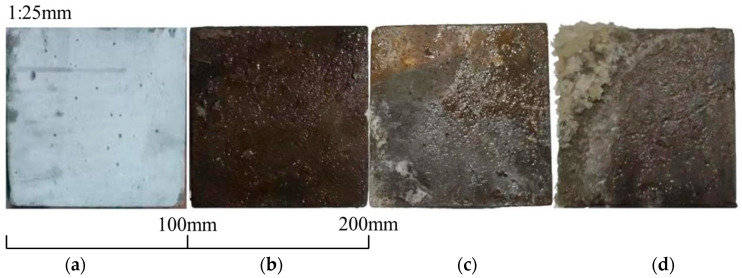
Gel precipitation of (**a**) C100, (**b**) R100, (**c**) B100, and (**d**) G100 (ordered from **left** to **right**) after soaking in 0.1 mol/L NaOH solution.

**Figure 3:**
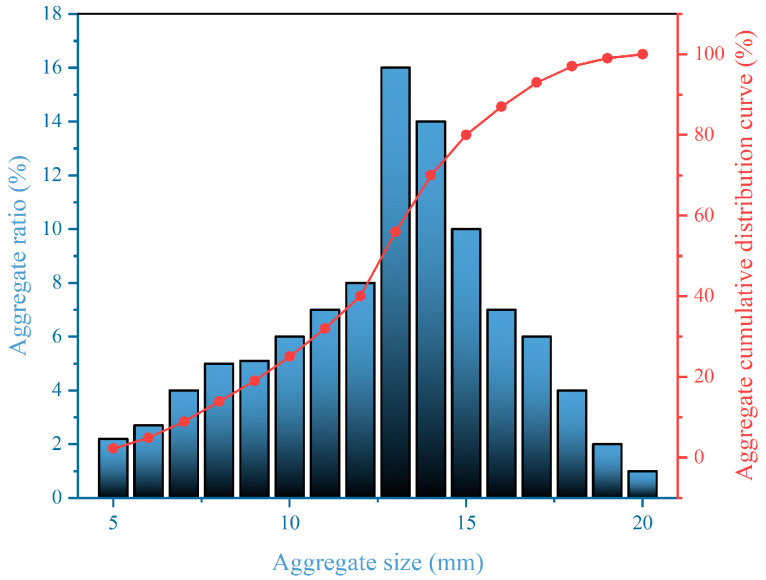
Particle size distribution curve of aggregate.

Concrete comprises water, cement, fine aggregate, coarse aggregate, and additional materials. The specimen for this study follows the C40 strength standard (C40 is a concrete strength grade with a required compressive strength of 40 MPa after 28 days of curing). For each group of concrete specimens, this study fabricated nine cubes (150 mm × 150 mm × 150 mm) and nine prisms (100 mm × 100 mm × 400 mm). Polypropylene fibers are blended with the aggregate and sand, and cement is introduced. The mixture is stirred, and water is added and thoroughly combined before being poured into molds. Utilized cement is low alkali cement to ensure the alkali content depends on the alkali–aggregate content. The fiber reinforcement used is polypropylene, which is 12 mm long and has a 0.91 g/m^3^ density [36]. Medium sand, having a fineness modulus of 2.4, serves as the fine aggregate. Four commonly utilized aggregates are selected to be susceptible to ASR and are blended in varying proportions to regulate the extent of ASR in concrete; their basic properties and composition are shown in Table 1 and Table 2. The dosage and the proportion of other raw materials are shown in Table 3. In this case, 30, 60, and 100 represent the alkali–aggregate content, and C, R, B, and G represent crushed stone made of pebble, recycled concrete, basalt, and granite. For example, R30 represents a concrete specimen with recycled concrete with a coarse aggregate content of 30%. Recycled concrete is crushed and screened with discarded concrete test blocks in the laboratory. Figure 1 shows the four chosen coarse aggregates: crushed stone, recycled concrete, basalt, and granite. Figure 2 illustrates that an increase in SiO_2_ in the coarse aggregate correlates with a higher propensity for these substances in other concrete raw materials to engage in ASR, resulting in more gel precipitation. This kind of gel is an alkali–silica gel. Figure 3 shows particle size distribution curve of aggregate. When the concrete has four conditions, including alkali aggregate, high alkaline solution content, soluble calcium, and specific humidity, ASR will occur, resulting in a chemical reaction between the alkaline solution and active silicon dioxide in the concrete, and finally, the formation of a gel. The reaction equation is as follows:

Na^+^(K^+^) + SiO_2_ + OH^−^→Na(K)—Si—H(gel) (1)

A more specific reaction process is divided into three steps. First, ions (Na^+^, K^+^, OH^−^, Ca^2+^) erode the aggregate, resulting in the dissolution of active SiO_2_; secondly, the silanol and siloxane bonds in active silicates are destroyed. Finally, basic silicates react with ions (Na^+^, K^+^, Ca^2+^) to form an alkali–silica gel.

This indicates that the SiO_2_ content in the aggregate, in order from high to low, should be basalt, granite, recycled concrete, and crushed stone. Therefore, under the same alkali–aggregate content, the higher the silica content of alkali aggregate, the greater the degree of alkali–aggregate reaction; for example, the extent of alkali–aggregate reaction of G30 is larger than that of B30. 

### 2.2. Mechanical Test

The pressure loading device and the mechanical control system complete the loading process of concrete test blocks. The pressure loading device adopts a YAM-5000F hydraulic testing machine (Beijing TIME High Technology Ltd., Beijing, China) to conduct the compressive strength and bending strength tests on the samples, with a loading speed of 0.3 MPa/s. 

### 2.3. Durability Test

In this study, our concrete specimens are subjected to two distinct and challenging conditions designed to test their durability and performance. The first condition is a 0.1 mol/L NaOH solution environment. The concrete groups containing alkali aggregate were left to rest in this solution for 28 days. The second condition is a series of freeze–thaw cycles (0, 25, 50 iterations). The freeze–thaw environment adheres to the specifications outlined in the “Standard for Test Methods of Long-term Performance and Durability of Ordinary Concrete” (GB/T 50082-2009 [37]). Each cycle spans 4 h, with the central temperature of the concrete specimen fluctuating between 8 ± 2 °C and −17 ± 2 °C. The freeze–thaw mediums employed are an aqueous solution. 

The damage sustained by concrete through different freeze–thaw cycles is assessed by monitoring shifts in their dynamic elastic modulus, mass, and strength loss rate. In this research, these parameters were measured per 25 freeze–thaw cycles. Blocks with varying alkali–aggregate types and ratios were subjected to specific experimental setups.

## 3. Damage Constitutive Model of Alkali Content in Polypropylene Fiber Concrete Based on Weibull Distribution 

### 3.1. Lemaitre Strain Equivalence

The deterioration process of concrete test specimens is intrinsically linked to their internal damage mechanisms. The material’s microstructure develops numerous micro-cracks subject to external loads, progressively evolving into macro-cracks. This progression leads to a decline in the concrete’s strength, stiffness, and toughness, thereby reducing its lifespan. Methodologies such as strain equivalence or strain energy assumptions are utilized to assess damage in the elastic state [38,39]. In this study, the strain equivalence hypothesis is adopted. Initially proposed by Lemaitre [40], the strain equivalence principle posits that the nominal stress exerted on the concrete equates to the effective strain of effective stress acting on an undamaged material. This approach introduces the damage variable D, whose underlying principle can be articulated as follows:

(2) $$\varepsilon =\frac{\sigma^{*}}{E\left(1-D\right)\;}=\frac{\sigma }{E}$$

where σ denotes the effective stress, D is conceptualized as the proportion of the area impacted by the loss of load-bearing capacity to the area unmarred by initial damage, and E signifies the concrete’s elastic modulus. The evolution of the concrete damage variable is categorized into two distinct phases. An increase in the damage variable concurrent with rising alkali–aggregate content defines the initial damage phase attributed to incorporating alkali aggregate. Drawing on damage mechanics theory [41], the macroscopic mechanical characteristics of concrete materials reflect the extent of internal degradation. Hence, for polypropylene fiber concrete with an alkali–aggregate substitution rate of r, its corresponding initial damage variable and initial elastic modulus is denoted as D_r_ and E_r_.

### 3.2. Parallel Bar System

**Figure 4:**
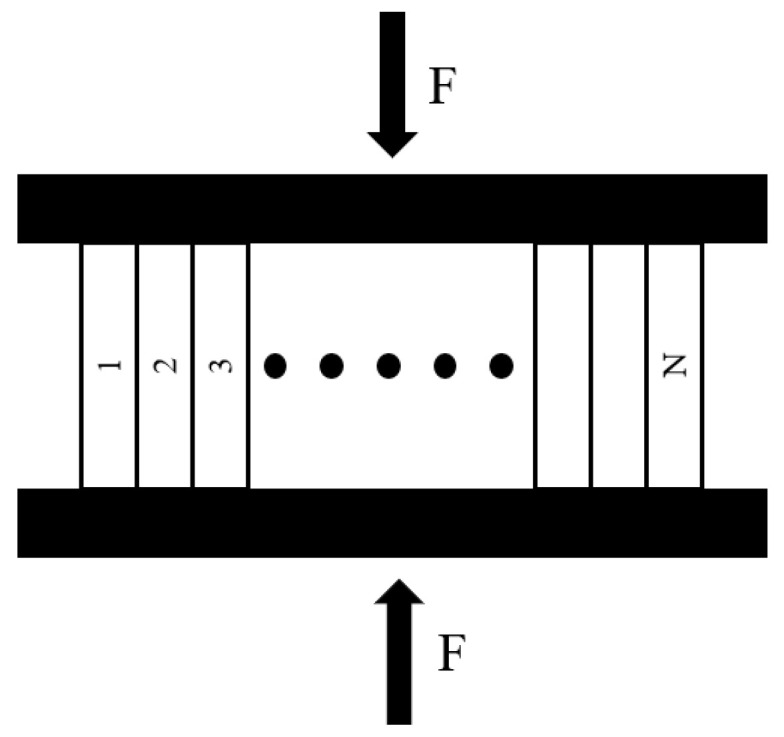
Parallel bar system.

As illustrated in Figure 4, the fine-scale statistical damage model is a key tool in our study. It is grounded on Krajcinovic’s simplistic mechanical parallel bar system. This model simulates the damage evolution in brittle materials like concrete and rock under the combined influence of internal stress redistribution and damage progression. The underlying concept of this model is to envision the concrete specimen as an assembly of countless micro-elements linked in series. Each micro-element is represented by elastic-brittle chain rods, which are parallel and uniformly spaced. The fracturing of these chain rods signifies the initiation of micro-damage. Although damage can occur randomly within each micro-element before the emergence of macroscopic cracks, the damage predominantly concentrates on the weaker surfaces after the formation of these cracks.

(3) $$A_{D}\left(\varepsilon \right)=\sum_{i=1}^{N}H\left(\varepsilon \;-\delta_{i}\right)dA_{i}$$

where: $\delta_{i}$ (i = 1, 2…N)—is the ultimate strain of the i-th rod unit; $dA_{i}$ (i = 1, 2…N) is the cross-sectional area of the i-th rod unit; N is the number of rod units in the PBS model; and Heaviside is the unit step function. 

Define the damage variable of the unit:

(4) $$D_{c}\left(\varepsilon \right)=\frac{A_{D}\left(\varepsilon \right)}{A}$$

Taking N → ∞, the unitary can be considered as a continuum, and D(ε) can be expressed as:

(5) $$\begin{array}{cc} D(\varepsilon ) & =\;\frac{A_{D}\left(\varepsilon \right)}{A}=\frac{1}{A}\left(\sum_{i=1}^{N}H\left(\varepsilon -\delta_{i}\right)dA_{i}\right)\\ & =\;\frac{1}{A}\left(\int_{0}^{A}H\left(\varepsilon -\delta_{i}\right)dA_{i}\right)\\ & =\int_{0}^{1}H\left(\varepsilon -\delta_{i}\right)d\left(A_{i}/A\right)\\ & =\int_{0}^{\varepsilon }f\left(x\right)\mathrm{dx} \end{array}$$

In this context, $f\left(x\right)$ represents the limiting strain of the rod unit, which adheres to a probability density distribution function. Consequently, the form of this distribution can be ascertained, allowing for determining the material damage variables.

The Weibull distribution, which integrates a chain model with statistical and probability theory, is a foundational failure theory. It is frequently utilized to represent the probability distribution of material failure, and its life prediction function finds extensive application across various domains. Numerous studies [42,43,44,45] have demonstrated that the probability distribution of concrete strength under uniaxial compression aligns with the Weibull strength theory. Specifically, the Weibull distribution is applied when evaluating the fatigue failure of polypropylene fiber concrete under an ASR. In this scenario, the degree of damage from the ASR is equated to the distribution function, with the expression presented as follows:

(6) $$F\left(\varepsilon_{R}\right)=1\;-\exp\left[-{\left(\frac{\varepsilon_{R}}{a}\right)}^{b}\right]$$

The damage variable $D_{c}$ can be expressed as follows:

(7) $$D_{c}\left(\varepsilon \right)=\int_{0}^{\varepsilon }p\left(\varepsilon \right)d_{s}=1-\frac{E_{r}}{E_{0}}\exp\left[-{\left(\frac{\varepsilon }{a}\right)}^{b}\right]$$

A fine-scale statistical damage model is employed to depict the intrinsic elastic-brittle structure of concrete, as detailed below.

(8) $$\sigma (\varepsilon )=E_{0}\varepsilon (1-D_{c}\left(\varepsilon )\right)=E_{0}\varepsilon \exp\left[-{\left(\frac{\varepsilon }{a}\right)}^{b}\right]$$

Based on the uniaxial compressive constitutive tests of concrete, the uniaxial compressive σ−ε curve exhibits a distinct peak stress, $\sigma_{t}$, and its corresponding strain, $\varepsilon_{t}$. This curve demonstrates a monotonic increase before the peak point and a subsequent decrease post-peak. The derivation of Equation (8) results in the following expression:

(9) $$\sigma^{’}\left(\varepsilon \right)=E_{r}(1\;-D_{c}(\varepsilon )\;-\varepsilon p(\varepsilon ))$$

At the peak point, where the slope is zero, the equation $\sigma^{\prime }(\varepsilon )$ = 0 possesses a unique non-zero solution. This solution represents the peak strain $\varepsilon_{t}$, corresponding to the peak stress $\sigma_{t}$. Its value is:

(10) $$\varepsilon_{t}={\;\varepsilon }_{0}{\left(\frac{1}{b}\right)}^{1/b}$$

The parameter b can be found by combining Equations (8) and (9):

(11) $$a=\frac{\varepsilon_{c}}{(1/b{)}^{\frac{1}{b}}}$$

(12) $$b={\;\left[\ln\left(E_{r}/\sigma_{c}\right)\right]}^{-1}$$

Drawing from the stress–strain curves acquired through concrete’s uniaxial compressive principal structure test, fundamental properties such as the modulus of elasticity $E_{r}$, peak stress $\varepsilon_{t}$, and peak strain $\sigma_{t}$ of polypropylene fiber concrete are determinable. Additionally, this enables the calculation of the parameters a and b.

**Figure 5:**
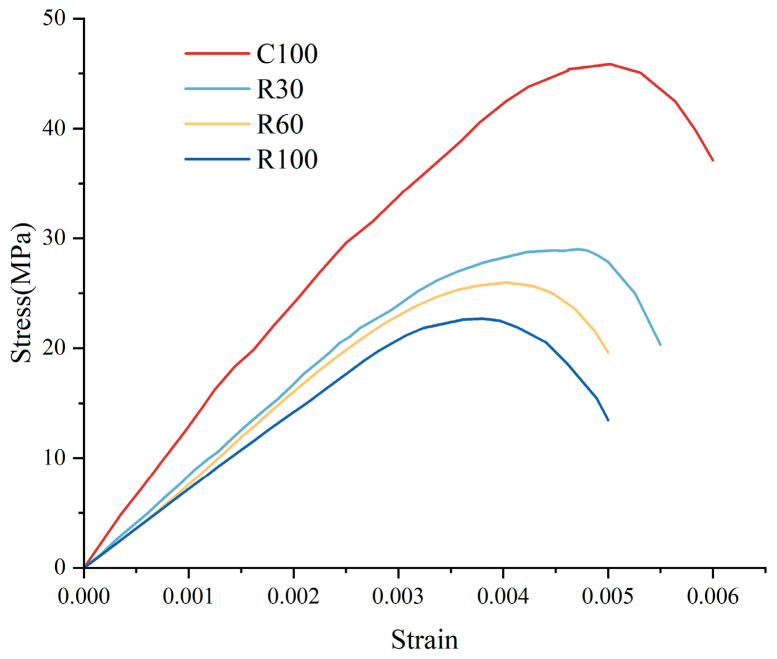
Uniaxial stress–strain curve of recycled concrete.

This investigation utilized experimental data obtained from C100, R30, R60, and R100 specimens to formulate the damage constitutive relationship about ASR. A pivotal aspect of this model is accurately determining the Weibull distribution’s shape parameters, denoted as a and b. The test specimens’ peak stress, strain, and initial elastic modulus, which are essential parameters for the model, were extracted from the experimental results and are graphically represented in Figure 5. 

The shape parameters a and b are derived using a specific mathematical formulation, as detailed in Equation (12). This equation is based on the tangent slope at 0.4 times the peak stress, a proxy for the concrete’s elastic modulus. The calculated values for these parameters are systematically compiled in Table 4, providing a comprehensive overview of the damage constitutive relationship for the different concrete mixtures studied. Establishing this relationship is crucial for predicting the long-term behavior and performance of concrete structures affected by ASR. By understanding the damage mechanisms and their progression, engineers and researchers can develop strategies to mitigate the impact of these reactions and enhance the durability and service life of concrete infrastructure. Using the Weibull distribution in this context offers a robust statistical framework for modeling the variability and uncertainty inherent in the material response to ASR.

**Figure 6:**
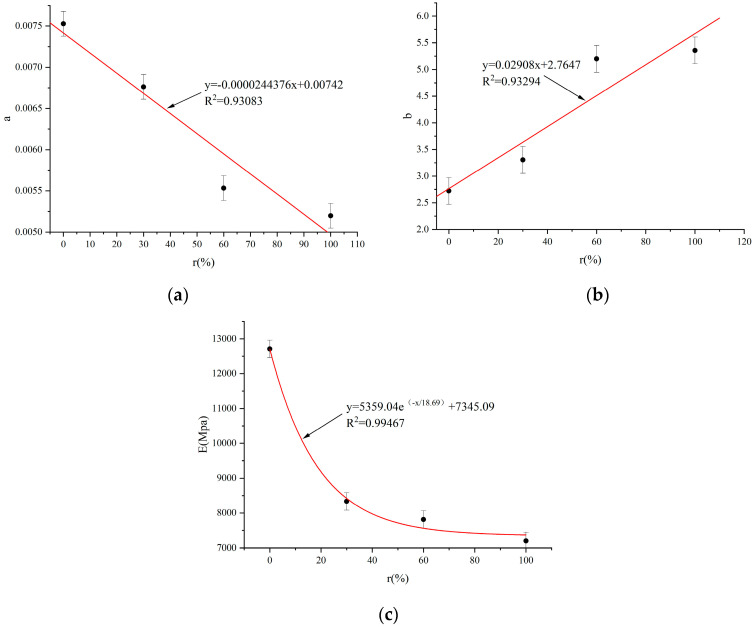
The relationship between parameters and alkali–aggregate content r: (**a**) variation of parameter a with alkali aggregate; (**b**) variation of parameter b with alkali aggregate; (**c**) variation of E with r alkali aggregate.

Utilizing the fitting function in Origin 2022 software, the relationship between the distribution parameters a and b, the elasticity modulus with alkali–aggregate content r was established, as depicted in Figure 6. It is observed that the correlation coefficients (R^2^) for all fitted curves exceed 0.9. This substantiates their capacity to accurately represent both the shape parameters and the relationship between the elastic modulus and r.

Previous research indicates that a smaller Weibull distribution parameter, a, correlates with reduced peak stress and strain. In contrast, a more significant parameter, b, is associated with decreased peak strain and increased brittleness [41]. Figure 6 shows that as r increases, both a and $E_{r}$ decrease, while b increases. This implies that higher alkali–aggregate content leads to lower peak stress and smaller peak strain in concrete, indicating increased brittleness. This observation aligns with the trends demonstrated by the experimental data in Figure 5. Equation (13) can be derived by substituting the related equations of a-r, b-r, and E-r.

(13) $$\sigma_{c}=((5359.04)exp((\frac{-x}{18.69})+7345.09)exp\left[-{\left(\frac{\varepsilon_{c}}{-0.0000244376x+0.00742}\right)}^{0.02908x+2.7647}\right]\;\;$$

**Figure 7:**
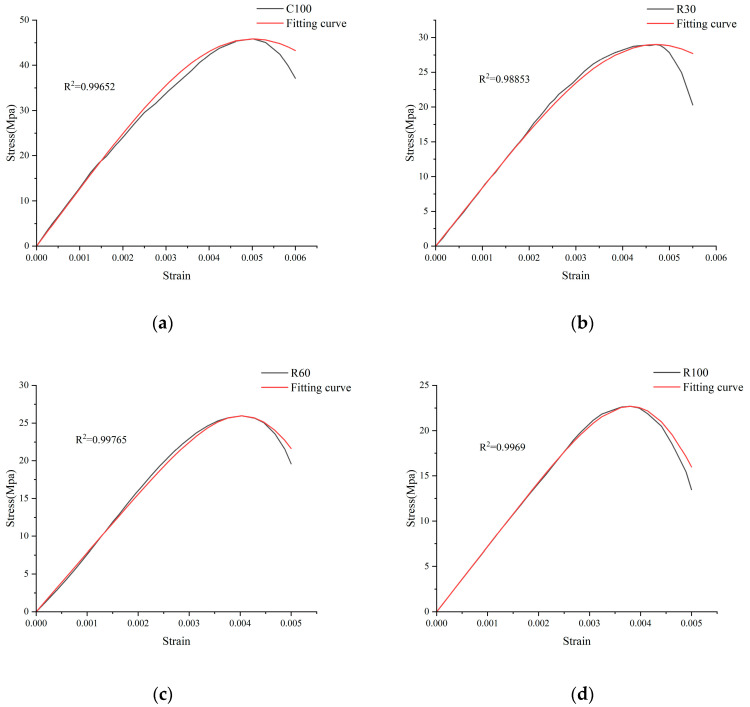
Test curve and theoretical calculation curve: (**a**) The fitting curve and C100 test curve; (**b**) The fitting curve and R30 test curve; (**c**) The fitting curve and R60 test curve; (**d**) The fitting curve and R100 test curve.

To verify the acceptable statistical damage intrinsic model of concrete proposed in this study, the theoretical fitting stress–strain curve is obtained by bringing the parameter data of Table 2 into Equation (13) and comparing the data with the stress–strain curve obtained from the test, as depicted in Figure 7.

Figure 7 illustrates that the alkali–aggregate damage model, as developed in this study, closely replicates the stress–strain characteristics of concrete when exposed to different concentrations of alkali–aggregate additives. The experimentally determined segments of the stress–strain curve, marked by an initial ascent followed by a subsequent descent, agree with the theoretical model proposed in this paper. The model’s accuracy is further supported by R^2^ exceeding 0.95, which signifies a minimal degree of dispersion between the empirical data and the theoretical predictions. This low variance indicates that the theoretical model precisely represents the concrete’s actual response to the alkali–aggregate additives. The high R^2^ value is a testament to the robustness of the model, suggesting that it can reliably predict the behavior of concrete under the influence of ASR. It is paramount for the construction industry, as it provides a valuable tool for assessing the durability and structural integrity of concrete structures that may be at risk due to alkali–aggregate additives. Moreover, the model’s predictive capabilities can be instrumental in guiding the design of concrete mixtures with enhanced resistance to ASR, thereby contributing to developing more sustainable and resilient infrastructure. By accurately simulating the stress–strain behavior of concrete, this model not only aids in understanding the underlying mechanisms of damage but also paves the way for implementing targeted mitigation strategies.

## 4. Results

### 4.1. Effect of Alkali Content on Mechanical Properties of Polypropylene Fiber Concrete

**Figure 8:**
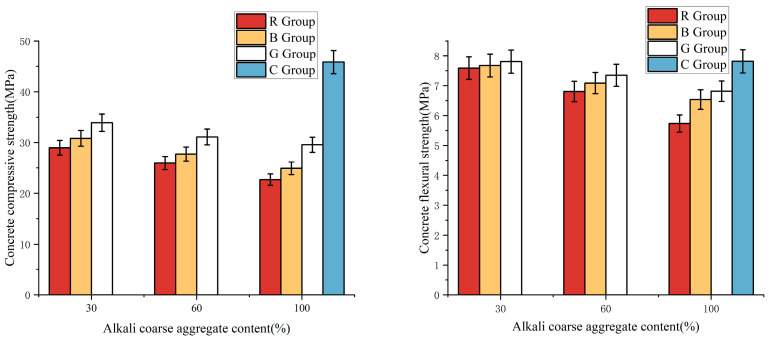
Concrete compressive and flexural strength of each group.

Table 5 illustrates the axial compressive and flexural strengths of concrete at different levels of alkali content. An increase in the alkali content of concrete is associated with a decrease in compressive and flexural strengths compared to the C100 sample, which does not contain additional alkali aggregate. This strength reduction is ascribed to the ASR. In this process, reactive silica in the alkali aggregate reacts with the alkalis present in the concrete, forming an alkali–silica gel. The absorption of water by this gel results in its expansion, which in turn triggers the formation of numerous microcracks throughout the concrete matrix, thereby reducing its structural integrity and load-bearing capacity. Figure 8 depicts an inverse correlation between the alkali content in concrete and its compressive and flexural strengths. A marked decrease in the concrete’s compressive and flexural strengths is observed with an escalation in alkali content.

### 4.2. Effect of Alkali Content on Durability of Polypropylene Fiber Concrete

#### 4.2.1. Mass and Relative Dynamic Elastic Modulus Loss Rate

Freeze–thaw cycles, a form of cyclic loading, induce a progressive transition in the internal structure of concrete from compact to loose, leading to the gradual accumulation of damage. Throughout this process, concrete’s mass and elastic modulus undergo continuous changes. Consequently, in evaluating the durability of concrete, its mass and elastic modulus are commonly employed as critical criteria. The test specimen’s mass and relative dynamic elastic modulus are measured after 25 freeze–thaw cycles. The changes in mass and relative dynamic elastic modulus of the test block are calculated in Equations (14) and (15).

(14) $$W=\frac{G_{0}-G_{1}}{G_{0}}$$

(15) $$D=\frac{E_{0}-E_{1}}{E_{0}}$$

where W is the loss rate of concrete mass; $G_{0}$ is the original mass of concrete before freeze thaw; $G_{1}$ is the mass of concrete after freeze thaw; $E_{0}$ is the initial elastic modulus; and $E_{1}$ is the dynamic elastic modulus after freeze–thaw damage.

**Figure 9:**
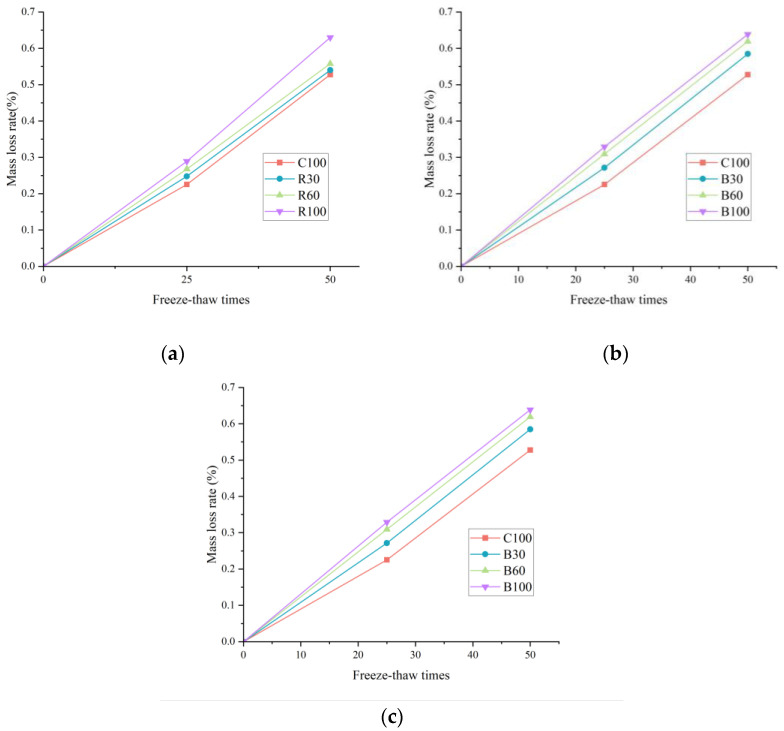
The mass loss rate of concrete: (**a**) mass loss rate of R group; (**b**) mass loss rate of B group; (**c**) mass loss rate of G group.

**Figure 10:**
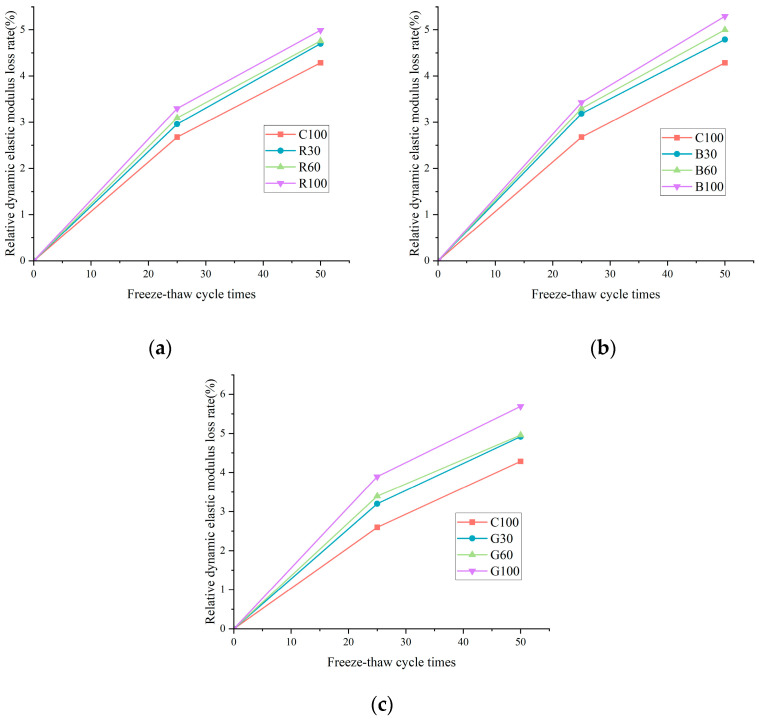
Relative dynamic elastic modulus loss rate of concrete: (**a**) relative dynamic elastic modulus loss rate of R group; (**b**) relative dynamic elastic modulus loss rate of B group; (**c**) relative dynamic elastic modulus loss rate of G group.

Figure 9 demonstrates a positive correlation between the increased alkali content, the corresponding mass loss rate increase, and a significant decrease in the dynamic elastic modulus under identical freeze–thaw cycling conditions. Notably, after 50 freeze–thaw cycles, the C100 sample recorded a mass loss rate of 0.53%, while samples R100, B100, and G100 showed higher rates of 0.63%, 0.64%, and 0.72%, respectively. It represents a relative increase of 18.87%, 20.75%, and 35.85% compared to the C100 sample. Similarly, Figure 10 indicates that the relative loss rate of the dynamic elastic modulus for C100 was 4.29% following 50 cycles. In contrast, the slightly elevated loss rates for R100, B100, and G100 were 4.99%, 5.30%, and 5.98%, respectively, which corresponds to increases of 16.32%, 23.54%, and 39.4% when compared to the C100 sample. These data underscore the detrimental impact of increased alkali content on concrete’s durability and structural integrity under cyclic environmental stressors such as freeze–thaw conditions.

The observed degradation in concrete subjected to freeze–thaw cycles is primarily due to its vulnerability to the forces of frost heave and osmotic pressure. Initially, these forces precipitate the formation of minute cracks on the concrete’s surface, which gradually extend and interconnect. Over time, these initial fissures develop into more substantial cracks, leading to the incremental disintegration and separation of the cement paste and fine aggregate from the surface. As the outer layer decays, new microcracks form and advance inwards on the newly exposed surfaces. This repetitive cycle leads to the outer cementitious layer’s erosion and an internal damage buildup, ultimately culminating in structural failure. Macroscopically, this process manifests as an increasing mass loss rate and a simultaneous reduction in the relative dynamic elastic modulus. Furthermore, the ASR exacerbates this deterioration by introducing additional microcracks, intensifying the damage from freeze–thaw exposure. This comprehensive understanding of the mechanisms at play underscores the importance of considering both the material’s resistance to such environmental conditions and the implications of its composition, particularly its alkali content, on its long-term performance and durability.

#### 4.2.2. Concrete Strength Loss Rate

As the primary indicator for assessing concrete durability, the residual strength rate post various freeze–thaw cycles was employed to analyze the influence of different alkali contents on concrete durability. This method facilitates a comparative evaluation of how varying alkali levels impact the concrete’s ability to maintain its structural integrity under freeze–thaw conditions.

(16) $$p=\frac{f_{0}-f_{1}}{f_{0}}$$

where p is the flexural strength loss rate of concrete; $f_{0}$ is the initial compressive strength of concrete without the freeze–thaw cycle; and $f_{1}$ is the compressive strength of concrete after the freeze–thaw cycle. 

**Figure 11:**
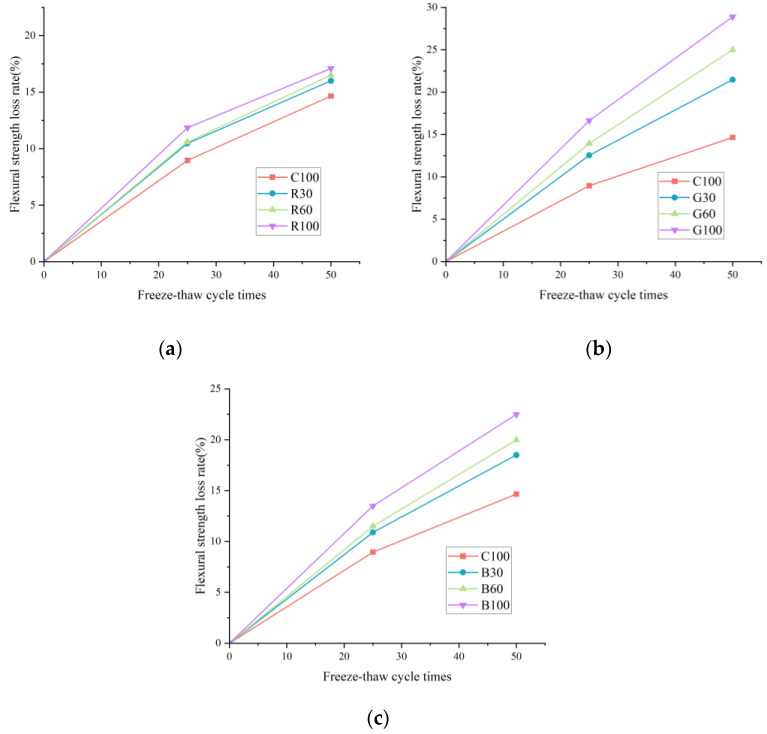
The strength loss rate of concrete: (**a**) strength loss rate of the R group; (**b**) strength loss rate of the B group; (**c**) strength loss rate of the G group.

Figure 11 presents data revealing that concrete experiences a more pronounced strength loss rate at lower levels of alkali content after a set number of freeze–thaw cycles. Specifically, the strength loss rate for the C100 sample was recorded at 14.65% after enduring 50 cycles, whereas the rates for R100, B100, and G100 were higher at 17.09%, 22.50%, and 28.90%, respectively. Compared to the C100 sample, these represent increases of 16.65%, 53.58%, and 97.28%, underscoring the negative influence of higher alkali content on the durability of polypropylene fiber concrete. The principal factor contributing to this decline in durability is the proliferation of micro-cracks stemming from the ASR, exacerbated during the freeze–thaw cycles. This progressive deterioration compromises the concrete’s load-bearing capability, substantially reducing the residual rates of flexural strength. The findings underscore the significance of alkali content in determining concrete’s long-term structural integrity and performance, particularly in environments subject to freeze–thaw cycles. 

**Figure 12:**
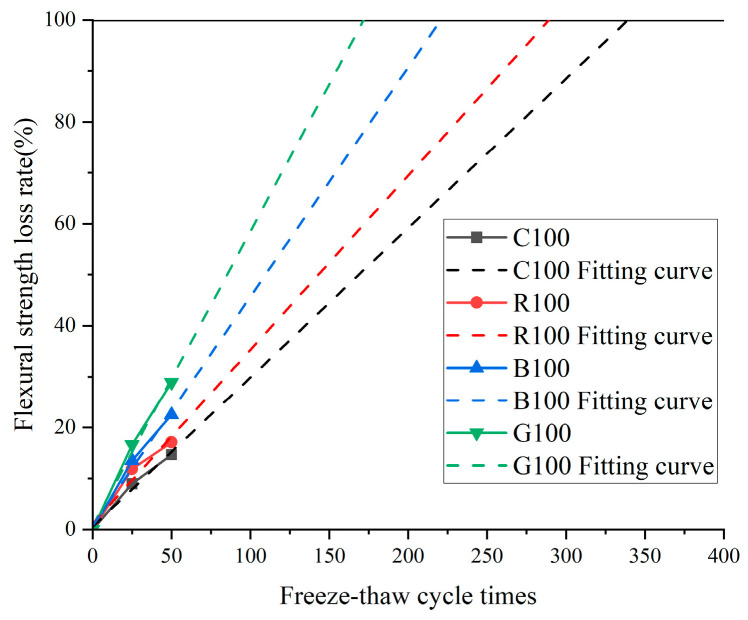
Flexural strength loss rate prediction curve at different freeze–cycle times.

Using Origin’s curve fitting capabilities allowed for a thorough analysis of the flexural strength loss rates, synthesized in Figure 12. The predicted number of freeze–thaw cycles necessary to complete strength depletion for the C100, R100, B100, and G100 samples are approximately 173, 221, 291, and 340 cycles, respectively. This observation, when considered from another angle, affirms that a positive correlation exists between higher alkali content and reduced durability of concrete. The findings underscore the critical role that alkali content plays in the longevity and resilience of concrete structures when subjected to the cyclical stresses of freeze–thaw cycles. The data indicate that concretes with lower alkali content exhibit more excellent resistance to strength deterioration under such conditions, thereby suggesting that careful management and reduction in alkali content could be instrumental in enhancing the durability of concrete and extending its service life.

## 5. Conclusions

This study investigates the impact of alkali content on the durability of polypropylene fiber concrete. It involves a comparative analysis of polypropylene fiber concrete’s mechanical properties and durability with varying alkali contents. Furthermore, the damage experienced by the concrete under uniaxial compression was simulated using the parallel rod model, leading to the establishment of a macroscopic damage model. The shape parameters of this model were determined based on the uniaxial compression test results, and the model’s rationality was subsequently validated. Conclusions are derived as follows:

(1) The constitutive damage model developed in this study is a testament to the precision of our research. It effectively captures the stress–strain relationship of concrete under various alkali contents, demonstrating high accuracy with a correlation coefficient greater than 0.95 compared to the experimental stress–strain curves. This model’s low dispersion and accurate reflection of real conditions instill confidence in its reliability.
(2) An increase in alkali content negatively impacts concrete’s bearing capacity and alters its mechanical properties. Specifically, concrete’s compressive and flexural strengths decrease to varying extents. For instance, when the alkali–aggregate content reaches 100%, the compressive and flexural strengths diminish by 35.5% and 32.9%, respectively.
(3) The adverse effects of alkali content on concrete’s durability are a cause for concern. With identical freeze–thaw cycles, an increase in the alkali content of concrete leads to a higher mass loss rate, a more significant reduction in relative dynamic elastic modulus, and a lower residual strength rate. Compared to concrete without alkali aggregate, the loss rates of mass, relative dynamic elastic modulus, and strength increase by 35.85%, 39.4%, and 97.28%, respectively, highlighting the detrimental impact of alkali content on concrete’s durability.

## Figures and Tables

**Table 1:** The basic characteristics of four types of coarse aggregates.

Aggregate Type	Apparent Density (kg/m^3^)	Water Absorption (%)	Hardness (Mohs)	Size (mm)
Crushed stone	2600	1.2	4.55	5–20
Recycled concrete	2500	6.0	4.54	
Basalt	2800	0.25	4.55	
Granite	2700	0.35	4.55	

**Table 2:** Composition of four types of coarse aggregate.

Aggregate Type	Composition	Granulometry
Crush stone	SiO_2_ (60–80%), CaCO_3_ (5–15%)_,_ Fe_2_O_3_ (1–5%)	The ratio of 5–10 mm coarse aggregate and 10–20 mm coarse aggregate is 3:7
Recycled concrete	Cement, Recycled aggregate	
Basalt	SiO_2_ (45–55%), Feldspar (20–30%), Mica (5–10%)	
Granite	SiO_2_ (70–80%), FeO (1–5%), MgO (1–4%)	

**Table 3:** Mix design of polypropylene fiber concrete (kg/m^3^).

Group	Water	Cement	Sand	Fiber	Crushed Stone	Recycled Concrete	Basalt	Granite
C100	195	487	613	0.91	1188	0	0	0
R30	195	487	613	0.91	831.6	356.4	0	0
R60	195	487	613	0.91	475.2	712.8	0	0
R100	195	487	613	0.91	0	1188	0	0
B30	195	487	613	0.91	831.6	0	356.4	0
B60	195	487	613	0.91	475.2	0	712.8	0
B100	195	487	613	0.91	0	0	1188	0
G30	195	487	613	0.91	831.6	0	0	356.4
G60	195	487	613	0.91	475.2	0	0	712.8
G100	195	487	613	0.91	0	0	0	1188

**Table 4:** Parameters of the principal structure equation of recycled concrete.

Group	Peak Strain	Peak Stress (MPa)	Elasticity Modulus (MPa)	a	b
C100	0.0052	45.86	12712	0.0075	2.72
R30	0.0047	29.00	8334	0.0068	3.30
R60	0.0040	25.98	7813	0.0055	5.20
R100	0.0038	22.70	7198	0.0052	5.36

**Table 5:** Strength of concrete at varying alkali content.

Group	Compressive Strength (MPa)	Flexural Strength (MPa)
C100	45.86	7.82
R30	29.00	7.59
R60	25.98	6.81
R100	22.69	5.74
B30	30.84	7.68
B60	27.75	7.09
B100	24.93	6.54
G30	33.92	7.81
G60	31.12	7.35
G100	29.58	6.82

## Data Availability

Data are available from the first author and can be shared with anyone upon reasonable request.
